# HDL‐Apolipoprotein in Alzheimer's Disease Revisited: From Periphery to CNS

**DOI:** 10.1002/agm2.70008

**Published:** 2025-03-20

**Authors:** Yihong Huang, Jingyi Tang, Guohua Chen, Qiangqiang Wu, Yongfei Wang, Jianjun Chen, Simei Chen, Jun Liu, Xiaoyun Huang

**Affiliations:** ^1^ Department of Neurology Houjie Hospital and Clinical College of Guangdong Medical University Dongguan Guangdong China; ^2^ Department of Neurology Sun Yat‐Sen Memorial Hospital of Sun Yat‐Sen University Guangzhou Guangdong China; ^3^ Department of Neurology The Second Affiliated Hospital of Guangzhou Medical University Guangzhou Guangdong China

**Keywords:** Alzheimer's disease, apolipoprotein AI, apolipoprotein E, high‐density lipoprotein

## Abstract

High‐density lipoprotein (HDL), as a crucial component of lipid metabolism, have roles in regulating Alzheimer's disease (AD) core pathology amyloid β (Aβ) and phosphorylated tau (p‐tau) through its apolipoproteins, which are associated with brain structures, cognition, and risk of dementia. The pool of HDL apolipoproteins—in the brain and in the periphery—has its own distinct origin, composition, and regulatory mechanisms. It remains unclear whether these apolipoproteins in the periphery and CNS play distinct roles in the pathogenesis of AD. Specifically, this review focus on the distinct associations of apolipoprotein AI and apolipoprotein E—the major components of HDL in the blood and CSF—with pathological proteins, brain integrity, cognition, and dementia progression in AD. We summarize and examine the current state of knowledge on the values of these apolipoproteins in AD pathogenesis and clinical potential.

## Graphical abstract

HDL‐apolipoproteins in the periphery and CNS contribute differently to Alzheimer's disease, influencing Aβ, p‐tau, brain structures, and cognition. Apolipoprotein AI and E, key HDL components in blood and CSF, may either protect against or accelerate AD, depending on their location. Created with BioGDP.com.

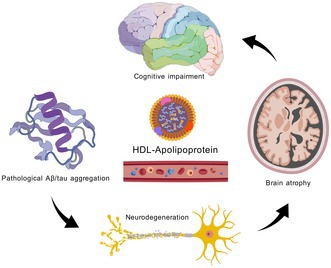

## 1 Introduction

As the most prevalent neurodegenerative disease, Alzheimer's disease (AD) progresses slowly, pathologically characterized by the loss of neurons, the deposition of amyloid‐beta (Aβ), and hyperphosphorylated tau (p‐tau), leading to progressive brain atrophy and cognitive decline. Evidence suggests dyslipidemia may appear and persist in the early stages of AD, interacting with pathological proteins like Aβ and p‐tau to initiate pathogenesis that mediate neuroinflammation, oxidative stress, and affect the vascular microenvironment and blood–brain barrier (BBB) integrity, ultimately leading to brain atrophy, and cognitive impairment [1, 2].

High‐density lipoprotein (HDL), an essential component of lipid metabolism, plays various critical roles in both periphery and central nervous systems (CNS), including cholesterol transport, protection against inflammation and oxidative stress, and promotion of endothelial integrity [3]. Importantly, the CNS relies heavily on the HDL system for lipid distribution [4, 5], making HDL system a significant area of research in aging and neurodegenerative disease. Numerous studies indicate that the protective effects of HDL in cardiovascular diseases are also prominent in AD, as higher levels of HDL are associated with lower Aβ deposition [6], less severe brain atrophy [7, 8], better cognitive function [9, 10, 11], and reduced risk of dementia progression [12]. However, recent studies have questioned the beneficial role of HDL levels in AD. Observational genetic studies have found that high plasma HDL levels may be associated with an increased risk of all‐caused dementia [13]. Mendelian randomization studies have not observed a causal relationship between HDL levels and AD [14, 15]. These findings suggest that the quality and function of HDL, rather than its levels, may be better predictors of AD. The quality and functionality of HDL largely depend on its composition, which is mainly abundant in apolipoproteins (Apos), including apolipoprotein A (ApoAI, ApoAII, ApoAIV), apolipoprotein C (ApoCI, ApoCII, ApoCIII), apolipoprotein D (ApoD), apolipoprotein E (ApoE), and apolipoprotein J (ApoJ, also known as clusterin, CLU) [2, 16]. Changes in the levels or nature of these HDL‐related apolipoproteins may affect the biological functions of HDL in regulation of diseases. Therefore, elucidating the roles of HDL‐related apolipoproteins in AD and targeting HDL functionality to improve clinical outcomes has become a burgeoning research direction.

There are differences in the composition of HDL in the periphery and CNS, with variations in the sources and biological functions of HDL‐related apolipoproteins. HDL in peripheral circulation is rich in ApoAI, while HDL in the CNS and cerebrospinal fluid (CSF) is mainly composed of ApoE [17, 18]. Peripheral ApoAI is primarily synthesized in the liver and not expressed in the CNS, potentially crossing the BBB from peripheral circulation into the CNS to perform cholesterol transport functions similar to those in peripheral tissues [5, 19]. Conversely, brain‐derived ApoE is primarily synthesized by glial cells or neurons, forming the major apolipoprotein in the CNS, separated from the peripheral circulation by the BBB [18, 20, 21]. The functional differences between ApoAI and ApoE in blood and cerebrospinal fluid (CSF) are not well studied, and it remains unclear whether these apolipoproteins in the periphery and CNS play distinct roles in the pathogenesis of Alzheimer's disease (AD). This review examines the differences between ApoAI and ApoE in blood and CSF, emphasizing their distinct associations with pathological proteins, brain integrity, cognition, and dementia progression in AD.

## 2 Apolipoproteins in Peripheral Blood and CNS

The pools of HDL in the brain and periphery are separated by different compositions and origins of apolipoproteins. While plasma HDL is enriched with ApoAI, HDL in the brain is primarily composed of ApoE, which coexist with ApoAII/IV, ApoCI/II/III, ApoD, ApoJ, and other proteins in both compartments [17, 18].

ApoAI is primarily synthesized and secreted into the bloodstream by liver and intestinal cells. It is absent in both murine and human primary brain endothelial cells, indicating that its presence in the CNS results from transport across the BBB and/or blood–CSF barrier (BCSFB) subsequent to its production by hepatocytes and enterocytes [5, 22]. Previous studies indicate that ApoAI entering the CNS from the periphery can facilitate the clearance of Aβ through the BBB [23, 24].

In the periphery, ApoE is primarily synthesized and secreted by liver cells, participating in the regulation of plasma lipid levels and maintaining lipid homeostasis in tissues. It is believed that peripheral ApoE is not able to cross the BBB, and its role in AD's pathology and brain structure remains unclear [25]. In the CNS, ApoE as the major apolipoprotein, is mainly synthesized and secreted by astrocytes and microglia, comprising the highest proportion of apolipoproteins in the CSF [5, 18].

As thus, the BBB and the BCSFB compartmentalize CNS apolipoprotein synthesis and metabolism separately from the periphery. The biological significance of plasma‐derived apolipoproteins in brain function and neurological disease progression, as distinct from mere reflection of CSF levels, remains uncertain. It is interesting to find out whether the neuromodulated properties are mediated by CNS derived apolipoproteins, through peripheral apolipoproteins entering the CNS from the periphery, or via both.

## 3 Link to Pathological Proteins of AD

### 3.1 ApoAI and Aβ

ApoAI has demonstrated significant interactions with Aβ in brain tissue. Immunohistochemical studies revealed the presence of ApoA1 in senile plaque, suggesting a potential cross‐interaction with Aβ [23]. ApoAI was found to bind to Aβ_1‐40_ and Aβ_1‐42_, inhibiting Aβ aggregation and facilitating Aβ clearance through the BBB, leading to the reduction of Aβ‐induced cytotoxicity, oxidative stress, and neurodegeneration [23, 24, 26, 27, 28]. In vitro models have demonstrated that the binding of ApoAI to Aβ disrupts the stability of Aβ fibrils, thereby facilitating their efflux across BBB [29]. In vivo animal models of AD have further validated the beneficial effects of elevated circulating ApoAI, including a reduction in cerebral Aβ deposition, attenuation of neuroinflammation, and inhibition of the aberrant activation of astrocytes and microglia [30, 31, 32]. Although the above research revealed the potential beneficial effect of ApoAI on Aβ, clinical studies did not observe correlation between CSF ApoAI and CSF Aβ [33] (Table 1). On the other hand, a study showed that plasma ApoAI was negatively correlated with the number of intracranial Aβ plaques measured by PET in cognitively normal elderly individuals [34] (Table 1). However, no correlation between plasma ApoAI and CSF Aβ was found in individuals with subjective cognitive decline (SCD), mild cognitive impairment (MCI), or AD [33, 35] (Table 1). Thus, while ApoAI in brain tissue may affect Aβ, the role of circulating ApoAI in blood and CSF in AD pathology remains unclear. The potential of circulating ApoAI as a predictive biomarker for AD pathology has yet to be explored.

**TABLE 1:** Studies of ApoAI and its associations with pathological markers, brain integrity, cognition and dementia progression.

Study	Subjects	Type of study	Soluble form	Association with Aβ	Association with t‐tau	Association with p‐tau	Association with brain integrity	Association with cognition	Association with dementia progression
Song et al. [36]	CN + MCI	Cross‐sectional; longitudinal	Plasma	/	/	/	/	No association	↓ Plasma ApoAI → ↑ risk of cogntive decline over 2 years
Hye et al. [37]	CN + MCIs+MCIc+AD	Cross‐sectional; longitudinal	Plasma	/	/	/	In AD group Mean hippocampal volume: *r* = −0.15, *p* = 0.04 Mean entorhinal thickness: *r* = −0.21, *p* = 0.01 Whole‐brain volume: *r* = −0.19, *p* = 0.02 In MCI group: NS	No association	No association
Shih et al. [38]	CN + AD	Cross‐sectional	Plasma	Plasma ApoAI can bind plasma Aβ	/	/	/	↑ Plasma ApoAI → ↑ MMSE (*r* = 0.51, *p* < 0.001)	/
Lin et al. [39]	CN + AD	Cross‐sectional	Plasma	/	/	/	/	↓ Plasma ApoAI → ↑ CDR	/
Westwood et al. [34]	CN	Cross‐sectional	Plasma	Plasma ApoAI showed negative association with PET Aβ load	/	/	/	/	/
Westwood et al. [35]	SCD + MCI + AD	Cross‐sectional	Plasma	No association	No association	No association	/	/	ApoAI were all nominally associated with MCI conversion to AD by logistic regression (*β* = 0.631, *p* < 0.05)
Slot et al. [33]	SCD + MCI	Cross‐sectional	Plasma+CSF	Plasma+CSF: No association	CSF: No association Plasma: *r* = 0.10, *p* < 0.05	Plasma+CSF: No association	/	/	↑ CSF ApoAI → ↑ risk of progression (attributable to the APOE e4 with SCD) ↓ Plasma → ↑ risk of progression (attributable to the APOE e4 with SCD)
Johansson et al. [40]	CN + MCI + AD+Other dementia	Cross‐sectional	Plasma+CSF	/	/	CSF ApoAI correlated negatively with CSF p‐tau (*r* = −0.25, *p* < 0.05)	/	CSF ApoAI correlated positively with MMSE score (*r* = 0.26, *p* < 0.05)	/
Romani et al. [41]	CN + AD+VaD	Cross‐sectional	Serum+CSF	/	Serum ApoAI: *r* = 0.266, *p* = 0.021	Serum ApoAI: *r* = 0.460, *p* = 0.003	/	/	/

### 3.2 ApoAI and Tau

Compared to its effects on Aβ pathology, the role of ApoAI in tau pathology and its related molecular mechanisms remains unclear. It is important to note that ApoAI may play an indirect role in the formation of NFT through its interaction with Aβ [24]. However, its direct impact on tau pathology requires further detailed in vivo and in vitro studies to be fully elucidated. On the other hand, only a few clinical studies have examined the correlation between circulating ApoAI and CSF tau protein, revealing differing associations between plasma and CSF ApoAI with CSF tau protein (Table 1). A previous analysis reported that the plasma ApoAI was positively correlated with CSF t‐tau and p‐tau, while the CSF ApoAI was negatively correlated with both biomarkers [42]. Slot et al. and Romani et al. found a positive correlation between plasma ApoAI and CSF p‐tau in patients with MCI and AD, respectively [33, 41]. However, another study involving individuals with varying degrees of cognitive impairment reported a negative correlation between CSF ApoAI and CSF p‐tau [40]. These discrepancies highlight the complexity and the need for further research to elucidate the precise role of circulating ApoAI in tau‐related processes in AD.

### 3.3 ApoE and Aβ

ApoE interacts with Aβ aggregates in intracranial senile plaques and cerebral amyloid angiopathy, influencing Aβ clearance through multiple pathways including enzymatic degradation, receptor‐mediated BBB transport, and glial cell phagocytosis [43]. The impact of ApoE on Aβ also depends on its protein isoforms. The ApoE4 isoform may exacerbate neurodegenerative processes by promoting Aβ formation and aggregation while hindering Aβ clearance, whereas the ApoE2 and ApoE3 isoforms exert neuroprotective effects by facilitating Aβ clearance [44]. Besides, neuronal ApoE receptors are likely involved in multiple processes [45], including the trafficking and processing of amyloid precursor protein (APP) [46], the clearance of Aβ [47], and the enhancement of Aβ synthesis through promoting the endocytic recycling of APP, with apoE4 being particularly implicated [48].

While several studies have failed to find a consistent association between CSF ApoE and Aβ pathology [49, 50, 51], positive correlations have been noted in specific demographic groups such as women and APOE ε4 carriers [52, 53], suggesting CSF ApoE may serve as a potential early marker for AD in these populations (Table 2). Conversely, for plasma ApoE, numerous clinical investigations have not identified a significant link with CSF Aβ levels [40, 49, 50, 51, 53] (Table 2). However, one study did reveal a decrease in plasma ApoE associated with reduced CSF Aβ across cognitive states even after adjusting for the influence of the APOE genotype, indicating a more intricate relationship that extends beyond the influence of APOE genotype alone [54] (Table 2).

**TABLE 2:** Studies of ApoE and its associations with pathological markers, brain integrity, cognition, and dementia progression.

Study	Subjects	Type of study	Soluble form	Association with Aβ	Association with t‐tau	Association with p‐tau	Association with brain integrity	Association with cognition	Association with dementia progression
Schmidt et al. [55]	AD	Cross‐sectional	CSF	/	/	/	/	No association with MMSE and other cognitive domain	/
Slot RER et al. [51]	SCD + MCI	Cross‐sectional	CSF	No association SCD + MCI: *β* = −0.05, *p* = 0.363 SCD: *β* = −0.02, *p* = 0.805 MCI: *β* = −0.03, *p* = 0.955	Positive association SCD + MCI: *β* = 0.49, *p* = 0.000 SCD: *β* = 0.65, *p* = 0.000 MCI: *β* = 0.44, *p* = 0.000	/	/	/	/
Liu et al. [52]	CN + MCI + AD	Cross‐sectional; longitudinal	CSF	Sex‐specific association Baseline All: *β* = 0.001, *p* = 0.5400 Female: *β* = 0.093, *p* = 0.0135 Male: *β* = −0.023, *p* = 0.3509 Longitudinal changes All: *β* = −0.002, *p* = 0.6880 Female: *β* = −0.018, *p* = 0.0104 Male: *β* = 0.006, *p* = 0.3131	Sex‐specific association Baseline All: *β* = 0.176, *p* = < 0.0001 Female: *β* = 0.170, *p* = < 0.0001 Male: *β* = 0.181, *p* = < 0.0001 Longitudinal changes All: *β* = 0.005, *p* = 0.2838 Female: *β* = 0.020, *p* = 0.0110 Male: *β* = −0.004, *p* = 0.5481	Sex‐specific association Baseline All: *β* = 0.102, *p* = < 0.0001 Female: *β* = 0.036, *p* = 0.3576 Male: *β* = 0.122, *p* = < 0.0001 Longitudinal changes All: *β* = 0.012, *p* = 0.1780 Female: *β* = 0.007, *p* = 0.6736 Male: *β* = 0.020, *p* = 0.0903	/	/	/
Hye et al. [37]	CN + MCIs+MCIc+AD	Cross‐sectional; longitudinal	Plasma	/	/	/	In AD group Mean hippocampal volume: *r* = −0.15, *p* = 0.05 Mean entorhinal volume: *r* = −0.18, *p* = 0.02 Mean entorhinal thickness: *r* = −0.20, *p* = 0.01 Whole‐brain volume: *r* = −0.19, *p* = 0.02 In MCI group: NS	In AD group MMSE: *r* = −0.150, *p* = 0.001 In MCI group MMSE: *r* = −0.150, *p* = 0.001 Longitudinal prospective MMSE change: NS in AD or MCI group	No association
Apostolova et al. [56]	MCI	Cross‐sectional	Plasma	/	/	/	/	/	Predicts progression to AD in participants with MCI over 36 months follow‐up
Koch et al. [57]	CN	Cross‐sectional	Plasma	/	/	/	/	↑ whole plasma apoE levels and ↑ apoE levels in HDL → better cognitive function assessed by ADAS‐cog (whole plasma, *β* coefficient, −0.15; 95% CI, −0.24 to −0.06; HDL, *β* coefficient, −0.20; 95% CI, −0.30 to −0.10) Higher apoE level in HDL that lacks apoC3 was associated with better cognitive function (ADAS‐cog per SD: *β* coefficient, 0.17; 95% CI, −0.27 to −0.07; Modified MMSE per SD: *β* coefficient, 0.25; 95% CI, 0.07–0.42)	Higher whole plasma apoE levels and higher apoE levels in HDL were unassociated with dementia or Alzheimer disease risk. Higher apoE level in HDL that lacks apoC3 was associated with lower risk of dementia (hazard ratio per SD, 0.86; 95% CI, 0.76–0.99).
Giannisis et al. [54]	CN + sMCI+cMCI+AD	Cross‐sectional; longitudinal	Plasma	Whole cohort: *ρ* = 0.340, *p* < 0.001 sMCI: *r* = 0.467, *p* = 0.009	Whole cohort: *ρ* = − 0.322, *p* < 0.001 cMCI: *r* = − 0.515, *p* = 0.005	Whole cohort: *ρ* = − 0.221, *p* = 0.016 cMCI: *r* = − 0.436, *p* = 0.020	/	Whole cohort: MMSE: *ρ* = 0.263, *p* = 0.003	/
Toledo et al. [49]	CN + MCI + AD	Cross‐sectional; longitudinal	Plasma+CSF	CSF and plasma ApoE: no association with basline levels or longitudinal changes of Aβ	CSF ApoE: in the whole cohort and non‐APOEε4 at baseline: ↑ CSF ApoE → ↑ CSF t‐tau no association with longitudinal changes of t‐tau; Plasma ApoE: no association	CSF ApoE: in the whole cohort and non‐APOEε4 at baseline: ↑ CSF ApoE → ↑ CSF p‐tau no association with longitudinal changes of p‐tau; Plasma ApoE: no association	↓ CSF ApoE → faster rate of atrophy in several cortical areas Plasma ApoE: no association	ADAS‐Cog longitudinal changes: CSF ApoE: *β* = −0.050, *p* = 0.0011 ↓ CSF ApoE → greater cognitive decline as measured by ADAS‐Cog Plasma ApoE: no association	MCI to AD conversion: CSF ApoE: ↑ CSF ApoE → ↓ risk HR = 0.70, *p* = 0.0086 Plasma ApoE: no association
Martínez‐Morillo et al. [53]	CN + AD	Cross‐sectional	Plasma+CSF	CSF ApoE: positive association in APOE ε4 carriers; Plasma ApoE: no association	CSF ApoE: positive association, especially ApoE isoforms 3 and 4; Plasma ApoE: no association	CSF ApoE: positive association, especially ApoE isoforms 3 and 4; Plasma ApoE: no association	/	Plasma and CSF ApoE: no association with MMSE	/
van Harten et al. [50]	SCD + MCI	Cross‐sectional; longitudinal	Plasma+CSF	CSF ApoE: no association Plasma ApoE: no association	CSF ApoE: positive association in APOEε4 carrier; Plasma ApoE: no association	CSF ApoE: positive association in APOEε4 carrier; Plasma ApoE: no association	/	↑ CSF ApoE → worse baseline performance on memory tests (*β* for immediate recall on the RAVLT: −1.04, *p* = 0.04; delayed recall of the RAVLT: −0.37, *p* = 0.04) No associations with cognitive decline over time. Plasma ApoE: no association	↑ CSF ApoE → ↑ risk of dementia progression in APOEε4 carrier HR = 1.5, *p* = 0.01 Plasma ApoE: no association
Johansson et al. [40]	CN + MCI + AD+Other dementia	Cross‐sectional	Plasma+CSF	CSF ApoE: positive association (whole cohort: *r* = 0.25, *p* < 0.05) CSF:plasma ApoE ratio: no association	CSF ApoE: positive association (whole cohort: *r* = 0.27, *p* < 0.05; AD cohort: *r* = 0.44, *p* < 0.05) CSF:plasma ApoE ratio: no association	CSF ApoE: positive association (whole cohort: *r* = 0.39, *p* < 0.001; AD cohort: *r* = 0.52, *p* < 0.01) CSF:plasma ApoE ratio: no association	/	CSF:plasma ApoE ratio: no association	/

### 3.4 ApoE and Tau

Regarding tau pathology, ApoE has been found to bind to receptors on the neuronal membrane, regulating tau protein phosphorylation and influencing the formation of neurofibrillary tangles (NFT) [58, 59]. It has been hypothesized that apoE isoforms may have distinct effects on tau pathology. In vitro studies showed that apoE3, but not apoE4, forms an SDS‐stable complex with tau, suggesting that apoE3 could potentially prevent abnormal tau hyperphosphorylation and the destabilization of the neuronal cytoskeleton [60]. On the other hand, the neuron‐specific effect of apoE4 on tau phosphorylation have been investigated in transgenic mice, which have demonstrated increased tau phosphorylation in neurons expressing human apoE4, but not in those expressing apoE4 in astrocytes [61, 62]. The differential contribution of apoE isoforms to tau hyperphosphorylation likely occurs through the modulation of tau kinases and phosphatases [45].

Several cross‐sectional studies involving CN, MCI, and AD populations have reported a positive correlation between CSF ApoE and CSF tau and p‐tau levels [40, 49, 50, 52, 53] (Table 2). It is speculated that the increase in CSF ApoE accompanying the rise in pathological tau proteins may be a protective response to neuronal damage. In terms of plasma ApoE, its association with CSF tau or p‐tau was not significant across several studies [40, 49, 50, 53] (Table 2). Only one study found that decrease in plasma ApoE was associated with an increase in CSF tau or p‐tau, indicating that a low plasma ApoE levels were unfavorably linked to AD pathology [54] (Table 2). It is evident that CSF ApoE levels are positively correlated with CSF tau and p‐tau levels, whereas plasma ApoE levels are negatively correlated with CSF tau and p‐tau levels. This suggests that ApoE in different tissues may play distinct roles in predicting changes in AD pathology, indicating functional heterogeneity of ApoE across tissues.

## 4 Link to Brain Integrity

Pathological Aβ deposition and tau phosphorylation further lead to neuronal degeneration and loss, manifesting as brain atrophy on structural magnetic resonance imaging (MRI), another hallmark of the AD disease spectrum. Research on the correlation between HDL‐Apos and brain atrophy is still limited, but there are still some clues (see Tables 1 and 2 for details).

### 4.1 ApoAI and Brain Integrity

Hye et al. found that in AD populations, plasma ApoAI levels are negatively correlated with the volume of the hippocampus, entorhinal cortex, and whole brain [37]. In contrast, Choi et al. discovered that in MCI populations, plasma ApoAI levels are positively correlated with the volume of brain regions such as the temporal lobe and cingulate gyrus [63]. Such contrasting results raise curiosity about whether plasma ApoAI has a beneficial or detrimental effect on brain structure. Additionally, these results only reflect cross‐sectional correlations, leaving the long‐term impact of plasma ApoAI levels on brain atrophy unknown. The impact of ApoAI on brain structure may be related to its role in Aβ regulation. ApoAI facilitates Aβ clearance through the BBB, potentially slowing neuron loss and brain atrophy. However, when pathological Aβ deposition leads to BBB dysfunction, impairing ApoAI's ability to clear Aβ, plasma ApoAI might adversely affect brain structure. Additionally, the inflammation and stress induced by pathological Aβ deposition may alter ApoAI's antioxidant and anti‐atherosclerotic properties, leading to vascular dysfunction, which is also associated with AD progression [64, 65, 66, 67, 68]. Despite the abovementioned research on plasma ApoAI and brain atrophy, studies on the correlation between CSF ApoAI and brain structure are still lacking.

### 4.2 ApoE and Brain Integrity

A cross‐sectional study found that increased plasma ApoE levels in AD patients are significantly associated with decreased hippocampal volume, reduced entorhinal cortex thickness, and enlarged ventricular volume [37]. Another longitudinal study conducted in CN, MCI, and AD populations did not find a correlation between plasma ApoE and brain atrophy, but decreased CSF ApoE levels were associated with accelerated gray matter atrophy. The inconsistency between the results of the above‐mentioned study may be related to the heterogeneity of the study populations. Additionally, the expression levels, protein isoforms, and biological functions of ApoE are controlled by the APOE genotype [43]. Research indicates that the ApoE4 isoform may exacerbate neurodegenerative processes by promoting Aβ formation and aggregation while hindering Aβ clearance, whereas the ApoE2 and ApoE3 isoforms may exert neuroprotective effects by facilitating Aβ clearance [44]. The impact of plasma and CSF ApoE on brain structure may also be influenced by ApoE expression levels, isoforms, and lipidation status [44], requiring further investigation.

## 5 Link to Cognition and Dementia Progression

In addition to its impact on pathological proteins and brain atrophy, whether HDL‐Apos correlates with cognition and clinical outcomes remains a key concern among clinical researchers. However, heterogeneity across clinical studies has prevented a unified consensus on the association between HDL‐Apos and cognition (see Tables 1 and 2 for details).

### 5.1 ApoAI and Cognition

Cross‐sectional studies have demonstrated a positive correlation between plasma ApoAI levels and Mini‐Mental State Examination (MMSE) scores [38], as well as a negative correlation with Clinical Dementia Rating (CDR) scores [39], suggesting a potential cognitive protective role for plasma ApoAI. However, other studies have indicated no association between plasma ApoAI levels and overall cognition or specific cognitive domains including memory, language, visuospatial skills, and executive function [36], thus limiting the clinical utility of plasma ApoAI as a predictor of cognitive function.

The different effects of ApoAI in plasma and CSF on cognitive changes have been previously reported. In line with our findings, Pillai et al. found that MCI or dementia patients with higher plasma ApoAI had a faster rate of cognitive decline, while those with higher CSF ApoAI had a slower rate of cognitive decline [69]. They speculated that such different effects might be related to the different associations of plasma and CSF ApoAI with BBB integrity and inflammatory markers [69]. This suggests that ApoAI in plasma and CSF may modulate AD‐related pathology in different ways, influencing clinical outcomes differently.

### 5.2 ApoAI and Dementia Progression

Plasma levels of ApoAI show a negative correlation or no association with dementia progression risk [33, 36, 56], indicating that ApoAI may possible protect against dementia progression. But for CSF ApoAI, higher levels were associated with increased risk of dementia progression in SCD patients carrying the APOE 𝜀4 allele [33]. Larger longitudinal studies are warranted to unveil the value of circulating ApoAI in predicting the risk of progression.

### 5.3 ApoE and Cognition

Research on plasma ApoE levels and cognitive function yields varied results. Koch et al. demonstrated a positive association between higher whole plasma ApoE levels and cognitive function, particularly assessed by ADAS‐cog, with higher ApoE levels in HDL also correlating positively [57]. Conversely, Giannisis et al. reported that lower plasma ApoE levels correlated with lower MMSE scores [54]. However, conflicting findings exist; some studies did not find any significant association between ApoE levels and cognitive function [53, 55], while others observed a negative correlation in certain contexts [37, 50]. These discrepancies highlight the complex and multifaceted relationship between plasma ApoE levels and cognitive performance, suggesting that additional factors may influence these associations across different study populations and methodologies.

For CSF ApoE, cross‐sectional studies found no correlation between CSF ApoE levels and cognition in populations with CN, MCI, or AD [40, 53, 55]. But another longitudinal study first reveal a decreased CSF ApoE levels were associated with a worse longitudinal outcome [49]. It is unclear why increased CSF ApoE levels might appear to protect against cognitive decline, but it is speculated that ApoE may be involved in injury repair mechanisms beyond Aβ amyloid plaques and tau NFT which linked to the cognitive changes [49, 70].

Attempts to investigate in detail the potential associations between circulating ApoE in blood or CSF and different cognitive domains are still limited. The above discrepant effects of ApoE in plasma and CSF may be partly explained by their different concentrations, origins, and functions in different compartments.

### 5.4 ApoE and Dementia Progression

Several studies have failed to find a correlation between plasma ApoE levels and the risk of dementia progression [37, 49, 50, 57]. In contrast, the association between CSF ApoE levels and dementia progression risk shows inconsistent results: some studies indicate that higher CSF ApoE levels were associated with increased dementia progression risk in individuals with APOEε4 allele in SCD or MCI [50], while others have found that lower CSF ApoE levels were associated with higher dementia progression risk in MCI [49]. Thus far, the correlation between HDL‐Apos and the risk of dementia progression lacks conclusive evidence, necessitating larger longitudinal cohort studies for further clarification.

## 6 Conclusion

Emerging evidence strongly supports the association between ApoAI and ApoE, key components of HDL particles in the periphery and CNS, with AD pathophysiology and clinical outcomes, suggesting a potential value of predicting and monitoring disease progression for HDL‐Apos. While data remain limited, the discrepancies effects of circulating ApoAI and ApoE in plasma or CSF on pathological proteins (Aβ, tau), brain integrity, cognition are not negligible. Future research on HDL‐Apos should consider their different sources and distributions, further exploring their exact roles in the peripheral or CNS during the development of AD. Targeting the regulation of HDL‐Apos in different tissues may enhance their vascular and neural protective functions, thereby intervening in the progression of the disease.

## Author Contributions

**Yihong Huang:** writing – original draft, writing – review and editing. **Jingyi Tang:** writing – original draft, writing – review and editing. **Guohua Chen:** writing – review and editing, investigation. **Qiangqiang Wu:** writing – review and editing, data curation. **Yongfei Wang:** writing – review and editing, visualization. **Jianjun Chen:** writing – review and editing. **Simei Chen:** writing – review and editing. **Jun Liu:** writing – review and editing, supervision, methodology, investigation, conceptualization, validation. **Xiaoyun Huang:** writing – review and editing, supervision, investigation, formal analysis, conceptualization.

## Ethics Statement

This review article adheres to the ethical standards of academic publishing. No new human or animal studies were conducted as part of this review. All sources referenced in this article were published and freely accessible to the public.

## Conflicts of Interest

The authors declare no conflicts of interest.
